# Spontaneous Multivessel Coronary Artery Dissection in a Middle-Aged Nigerian Male Patient With Chest Pain: A Case Report From Nigeria

**DOI:** 10.7759/cureus.115682

**Published:** 2026-09-02

**Authors:** Olurotimi J Badero, Jennifer C Okei, Victor Ajayi, Favour E Godgift, Nwachukwu O Nwachukwu, Ifeoluwa S Bakare, Bamikole Osibowale

**Affiliations:** 1 Interventional Cardiology, Iwosan Lagoon Hospital, Lagos, NGA; 2 Cardio-Nephrology, Cardiac Renal and Vascular Associates, Jackson, USA; 3 Medicine, College of Medicine, University of Lagos, Lagos, NGA; 4 Surgery, University of Lagos, Lagos, NGA; 5 Medicine, Lagos State University Teaching Hospital, Lagos, NGA; 6 Surgery, Gregory University Uturu, Amaidi, NGA; 7 Public Health, Liverpool John Moores University, Liverpool, GBR; 8 General Medicine, Ternopil State Medical University, Ternopil, UKR; 9 Cardiology, Caribbean Tristate Heart Institute, Trinidad, TTO

**Keywords:** chest pain, coronary artery disease, coronary artery dissection, nigeria, west africa

## Abstract

Spontaneous coronary artery dissection (SCAD) is now more frequently recognized as an etiology of acute coronary syndrome, primarily reported in women. Its occurrence in men remains rare and often goes unnoticed and underreported, especially in low- and middle-income countries like Nigeria, where limited access to advanced coronary imaging and low clinical suspicion hinder diagnosis.

A 46-year-old Nigerian man was admitted with acute chest discomfort, T-wave abnormalities on electrocardiography and normal cardiac troponins. Coronary angiography showed multivessel spontaneous coronary artery dissection (SCAD) involving left anterior descending (LAD) and left circumflex (LCX) arteries. He was treated conservatively with good clinical outcome.

SCAD should be considered in men with acute coronary symptoms, even when traditional cardiovascular risk factors are present. In Nigeria and similar resource-limited settings, maintaining a high level of suspicion and improving access to advanced cardiac diagnostics are essential for overcoming diagnostic challenges and providing appropriate, non-invasive treatment. To the best of our knowledge, following a comprehensive search of published literature, this represents the first described case of SCAD in a Nigerian male patient and one of the earliest cases documented in West Africa. The aim of this case report is to increase local awareness and promote a high index of suspicion for SCAD in men presenting with acute coronary syndrome, particularly in resource-limited settings.

## Introduction

Spontaneous coronary artery dissection (SCAD) is an entity involving non-traumatic, non-iatrogenic, and non-atherosclerotic separation of the layers of an epicardial coronary artery. This results from an intramural hematoma leading to myocardial injury [1,2]. SCAD is a major etiology of myocardial infarction (MI) with non-obstructive coronary arteries (MINOCA), contributing about 20% of such cases and 1%-4% of acute coronary syndrome events [3,4]. Risk factors associated with SCAD include genetic predisposition, female gender, arteriopathies such as fibromuscular dysplasia, hormonal states such as pregnancy, and systemic inflammatory diseases [1,5]. Current evidence shows that men are less likely to have SCAD, accounting for about 10% of presentations [6]. 

SCAD is classified by its angiographic appearance into four main types: Type 1 shows classic arterial wall contrast staining with multiple radiolucent lumens, Type 2 presents as diffuse smooth arterial narrowing with varying severity, Type 3 mimics focal atherosclerosis, and Type 4 causes an abrupt total vessel occlusion usually involving distal segment of the artery [7]. Diagnosing SCAD in resource-limited settings is highly challenging largely due to limited access to advanced coronary imaging modalities. Furthermore, its presentation may not differ from that of acute coronary syndrome of atherosclerotic aetiology, particularly in male patients with traditional cardiovascular risk factors.

There has been no previously documented case of multivessel SCAD in males in this geographical population to the best of our knowledge. Very few cases have been reported in Africa, which may be due to low publication rates or underdiagnosis from limited access to advanced coronary diagnostic studies.

## Case presentation

**Figure 1:**
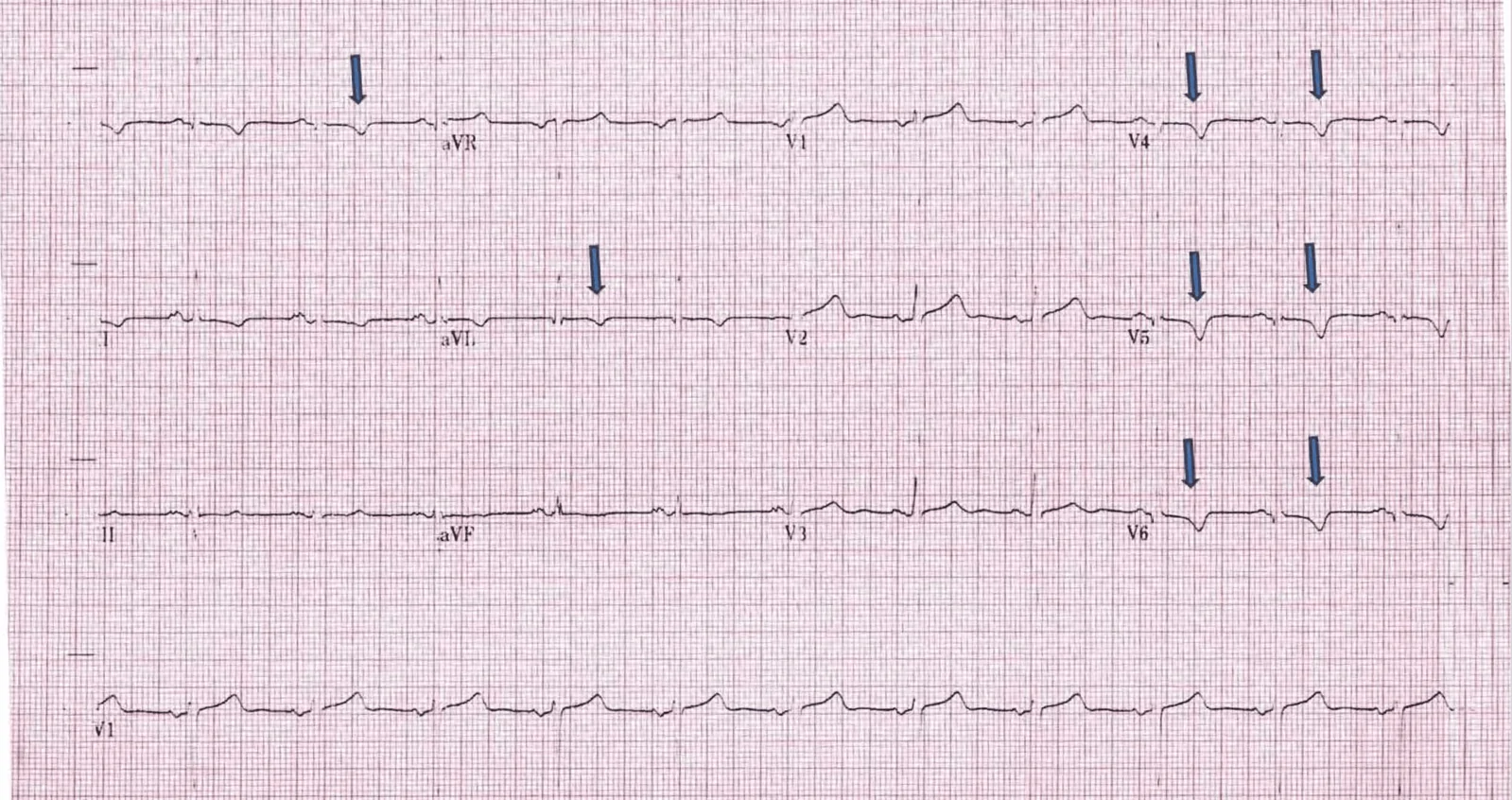
Twelve-lead ECG on presentation showing T-wave inversion in leads 1, AVL, V4, V5, and V6 (blue arrows) AVL: Augmented vector left.

The case is that of a 46-year-old Nigerian man who had a previous history of hypertension, hyperlipidemia, and peripheral arterial disease (PAD) complaining of exertional chest pain and dyspnea. His condition had gradually worsened, with his pain symptoms now occurring at rest. His father also had coronary artery disease. Alcohol and tobacco use history was negative. His outpatient medications included amlodipine 10 mg once daily, hydrochlorothiazide 12.5 mg once daily, atorvastatin 20 mg nocte, and aspirin 75 mg once daily, which he has been taking consistently for the six months before presentation. On presentation, his vital signs were as follows: pulse rate was 66 beats per minute, blood pressure was 136/97 mmHg, respiratory rate was 18 breaths per minute, and oxygen saturation was 99% on room air. Electrocardiogram showed normal sinus rhythm with T-wave inversion in the lateral, anterior, and antero-lateral leads, consistent with ischemia in the left circumflex artery (LCX) and left anterior descending artery (LAD) territories, respectively (Figure 1).

Serial troponin levels and kidney function tests were normal. His lipid profile revealed a total cholesterol level of 186 mg/dl (desirable <200 mg/dl), LDL-cholesterol was 106.7 mg/dl (optimal <100 mg/dl), HDL-cholesterol was 48 mg/dl (normal >40 mg/dl in men) and triglycerides was 156.5 mg/dl (normal<150 mg/dl).

Echocardiography demonstrated a normal left ventricular geometry, and systolic function (ejection fraction: 69%), left ventricular diastolic function and right ventricular systolic function were normal. Valvular function was normal. There were no regional wall motion abnormalities. A diagnosis of unstable angina was made with an intermediate score of 3 using the thrombolysis in myocardial infarction (TIMI) risk score.

**Figure 2:**
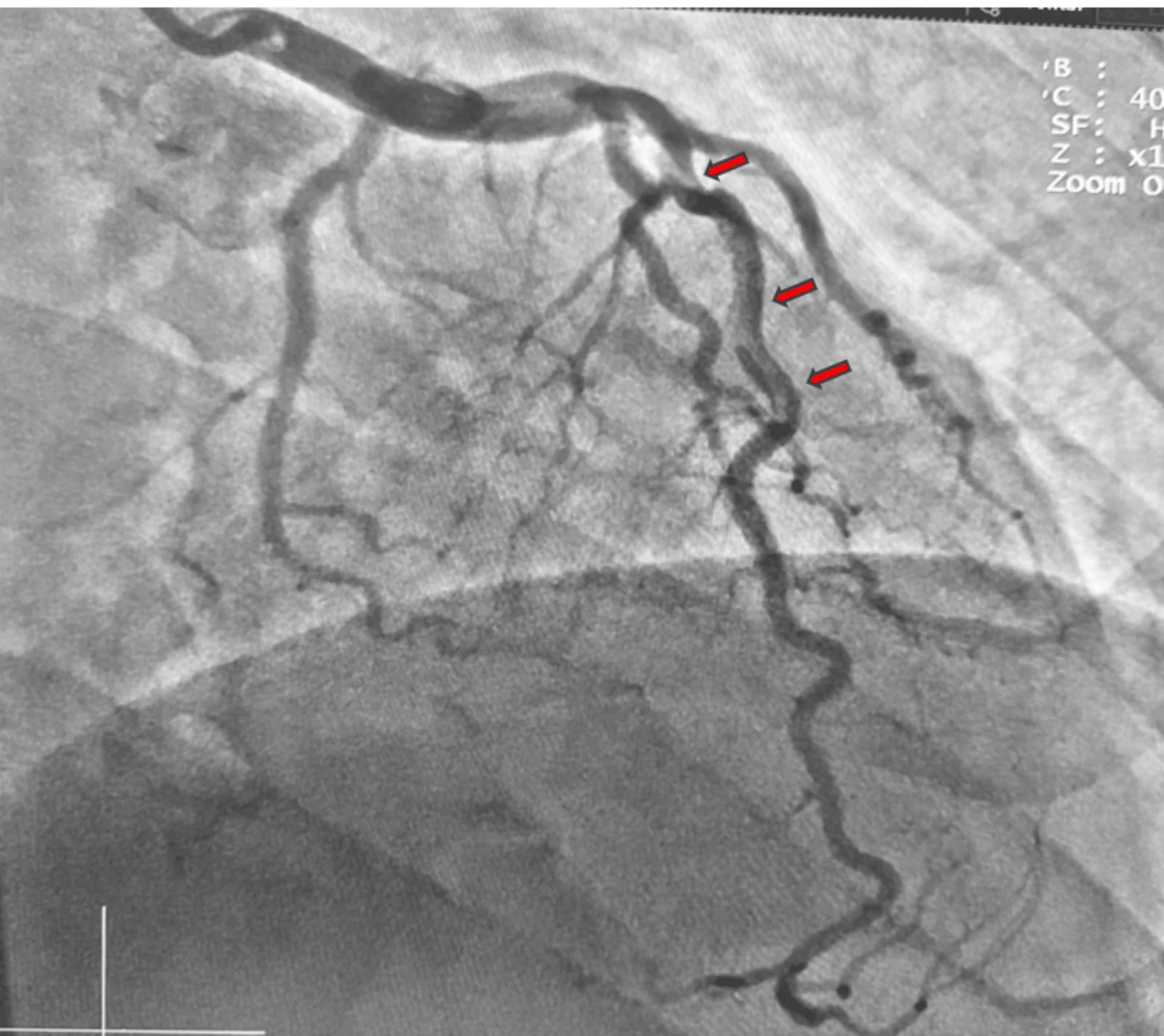
Coronary angiogram showing spontaneous coronary artery dissection (SCAD) involving the mid-left anterior descending coronary artery (red arrows).

**Figure 3:**
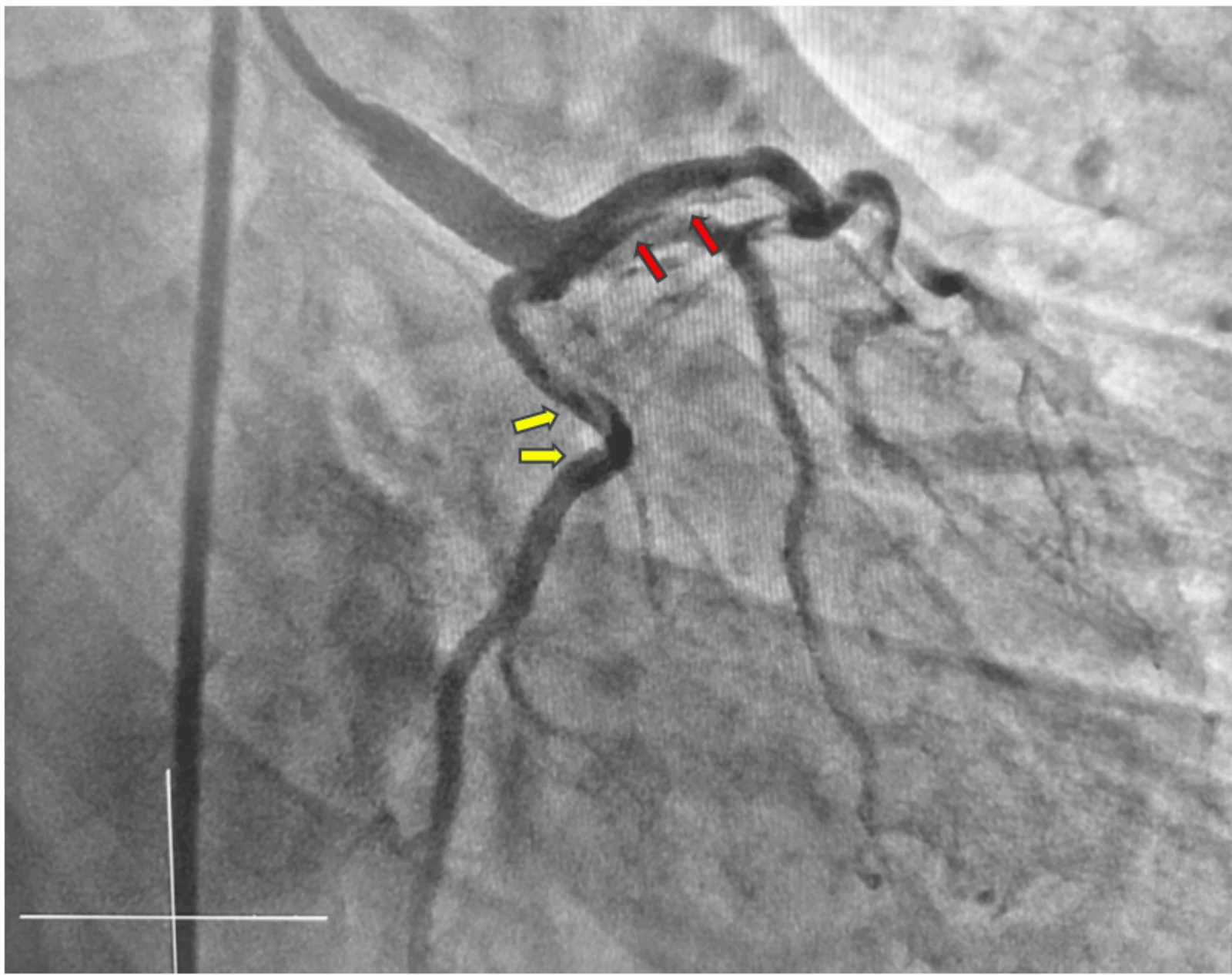
Coronary angiogram showing spontaneous coronary artery dissection (SCAD) involving the left circumflex coronary artery (yellow arrow) and proximal left anterior descending coronary artery (red arrows).

**Figure 4:**
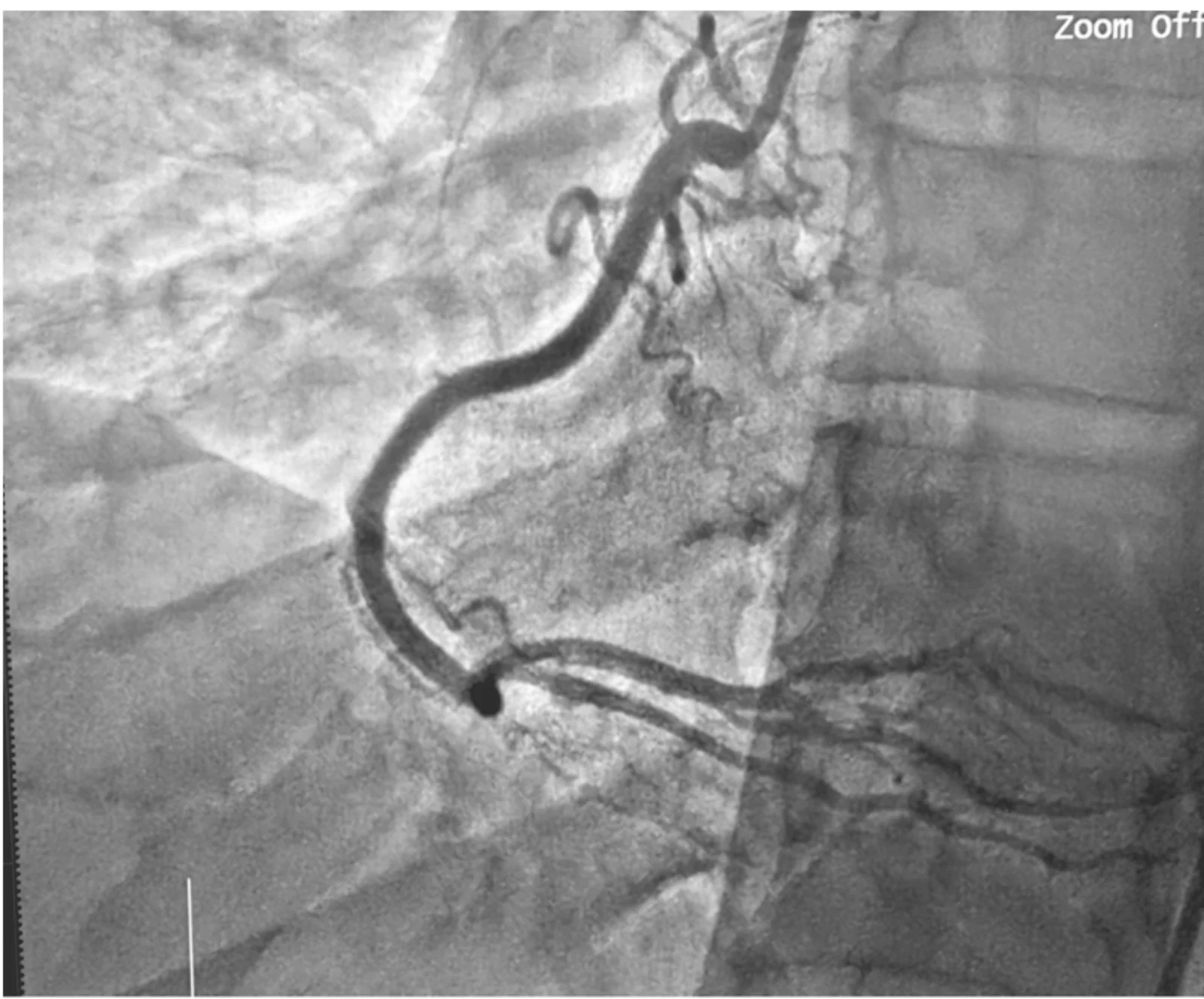
Coronary angiogram showing the right coronary artery without spontaneous coronary artery dissection (SCAD).

He was initiated on metoprolol succinate 25 mg once daily, continued on atorvastatin 20 mg nocte, amlodipine 10 mg once daily and hydrochlorothiazide 12.5 mg once daily with a planned cardiac catheterization within 24-72 hours using a delayed invasive strategy. Coronary angiogram obtained showed angiographically normal left main coronary with type 1 SCAD involving the proximal and mid LAD and the proximal segment of the LCX with TIMI 3 flow (Figures 2, 3). Right coronary artery was free of any significant disease (Figure 4). Notably, he had chest pain with each contrast injection into the left coronary system but not the right. Intravascular imaging was not an option in this case due to non-availability. He was managed medically with aspirin 81 mg once daily and metoprolol 25 mg once daily given his hemodynamic stability, lack of ventricular arrhythmia, and absence of left main coronary artery disease.

He remained hemodynamically stable and chest pain-free on day 4 of admission and was subsequently discharged. No recurrent symptoms were reported at a four-week follow-up.

## Discussion

SCAD is an underlying etiology of acute coronary syndrome marked by the formation of intramural hematoma secondary to bleeding from the vasa vasorum. It involves the separation between the intima, media, and adventitia layers of the coronary artery wall. As a result, a thrombus-containing false lumen forms, which is neither iatrogenic nor associated with atherosclerosis [5,8]. SCAD causes acute coronary syndrome through pressure-driven expansion of an intramural hematoma, formed by the accumulation of blood or clot within the vessel wall. This expansion propagates the false lumen tear, while compressing the true lumen, thereby leading to myocardial infarction. Dissections are typically more extensive, with unaffected coronary arteries appearing smooth and free of atherosclerotic disease on cardiac catheterization, as seen in our patient [5,9].

Many cases of SCAD are idiopathic. However, some conditions that increase wall stress can raise the risk of SCAD. Studies indicate that physical triggers are more common in men, while physiological stress triggers are more commonly observed in women [10]. Other common associations include arteriopathy, connective tissue disorders, pregnancy, and peripartum states.

The presentation and severity vary. Symptoms may include weakness, dizziness, headaches, chest pain with no electrocardiogram changes, and there may be ST-segment or T-wave changes with accompanying elevated cardiac injury biomarkers. Sometimes, it is an incidental finding during coronary computed tomography angiography (CCTA) or cardiac catheterization [5]. Women have a higher likelihood of presenting with chest pain along with symptoms such as neck pain, jaw pain, back discomfort, and shortness of breath. Men, in contrast, tend to present solely with chest pain [10].

Because its angiographic features are often underrecognized and the condition underdiagnosed, estimating the true prevalence of SCAD is challenging, especially in men, who are less frequently affected. As far as we are aware, this represents the first documented SCAD case in a Nigerian male patient, as existing literature reports a few cases in Nigeria, with none documented in men, and only a single case reported in West Africa [11]. Most cases are recorded in women, with a higher percentage (60%-75%) involving the left anterior descending (LAD) artery. Other affected sites include the left main coronary artery (LMCA), left circumflex (LCX) artery, and sometimes multiple coronary arteries, as in our case [5,12]. SCAD differs notably between women and men, with women accounting for most cases. They often present with Type 2 SCAD, characterized by long, diffuse coronary narrowing due to intramural hematoma, usually involving mid-to-distal segments and often associated with fibromuscular dysplasia and hormonal factors [1,13]. In contrast, SCAD in men is less common, tends to occur at an older age, is often triggered by physical exertion, and more frequently presents as Type 1 SCAD, with visible intimal disruption and proximal coronary involvement [1,6].

Estrogen-associated effects on connective tissue and microvascular integrity may predispose women to intramural hemorrhage, while greater exposure to mechanical shear stress and the lack of hormonal vascular protection in men may favor intimal tearing [1,14]. These factors may contribute to the observed differences in SCAD type and coronary locations between genders. Gender-related differences in the anatomical distribution of SCAD are limited and not consistently defined. The findings from the Canadian SCAD Study suggest that while overall angiographic patterns are similar, involvement of the left circumflex artery may be more common in men and could explain less typical electrocardiographic presentations [6,12]. Conversely, larger international registries have found no significant sex-based differences in the affected coronary territories or in the occurrence of multivessel SCAD, highlighting the substantial overlap in disease patterns between sexes [10].

In our patient, both the LAD and LCX arteries were affected, consistent with reported patterns in the literature on male SCAD. This case illustrates the heterogeneity of SCAD anatomy, emphasizing the critical value of considering SCAD as a potential etiology of acute coronary syndrome in men. SCAD diagnosis can be supported by several imaging modalities. While invasive coronary angiography remains the gold standard for diagnosing SCAD, it carries an inherent risk of further propagating the dissection [15]. In challenging cases with uncertain diagnosis, intravascular ultrasound (IVUS) or optical coherence tomography can provide a clearer visualisation of coronary arterial wall layers to identify disruption or hematoma [16]. Due to the risk of dissection propagation associated with invasive coronary angiography, CCTA is increasingly being used as a substitute in stable patients with suspected SCAD. It provides a non-invasive approach with a high spatial and temporal resolution [17]. Cardiovascular magnetic resonance (CMR) can provide a detailed assessment of cardiac anatomy, function, and perfusion with sufficient spatial resolution for SCAD evaluation, while avoiding exposure to ionizing radiation [18].

Treatment of SCAD is generally tailored to the patient. Asymptomatic patients are managed conservatively with risk factor control. Symptomatic patients may require coronary stenting or surgery for vessels with significant stenosis [19]. Pharmacologic therapy after percutaneous coronary intervention (PCI) typically includes dual antiplatelet therapy, beta blockers, calcium channel blockers, statins and renin-angiotensin system antagonists, with antiplatelets and beta-blockers serving as the mainstay of conservative management. Anticoagulation is generally not recommended in SCAD management [20]. Our patient was managed conservatively with a good clinical outcome. Several studies have demonstrated no significant gender-based differences in major adverse cardiovascular outcomes (MACE), including recurrent SCAD, stroke, non-fatal myocardial infarction, and SCAD-related mortality [21,22].

## Conclusions

SCAD is an uncommon yet clinically relevant etiology of acute coronary syndrome, particularly in men, where it is often underrecognized. Our report describes an unusual presentation of SCAD in a Nigerian man. Prompt recognition and conservative treatment approach with guideline-directed medical therapy can lead to favorable outcomes while avoiding unnecessary invasive interventions. This case underscores the importance of raising awareness, improving reporting, and conducting further research on SCAD in underrepresented populations.
